# Public Play Grounds

**Published:** 1913-11

**Authors:** 


Public Play Grounds. According to the *Survey*, Birmingham, Ala., is the last of the cities of the U. S. of over 100,000 to provide public play grounds. We are grateful, personally, to have lived when there were plenty of vacant lots and large yards in which to play ball, hunt bears, bury dead cats and build bonfires, even though there were always some impatient to put them out before the potatoes were roasted. and we are doubly grateful that our play was without modern contrivances and not personally conducted. But times change, and now that we must have public play grounds, let us have enough of them at convenient intervals to do away with the necessity of making the streets play grounds also, playing in the street costs too many lives and produces too much nerve strain of conscientious drivers.

